# Research Note: Comparative effects of liquid and dry applications of a combination of lysolecithin, synthetic emulsifier, and monoglycerides on growth performance, nutrient digestibility, and litter moisture in broilers fed diets of differing energy density

**DOI:** 10.1016/j.psj.2023.103345

**Published:** 2023-12-03

**Authors:** Alexandra L. Wealleans, Alexandra Desbruslais, Rita Goncalves, Dawn Scholey, David Gonzalez-Sanchez, Emily Burton, Riet Spaepen, Allison Elliott, Douglas Currie

**Affiliations:** ⁎ Kemin Animal Nutrition and Health, Kemin Europa N.V., 2200 Herentals, Belgium; † Roslin Nutrition Ltd., Aberlady, United Kingdom; ‡ School of Animal, Rural and Environmental Sciences, Nottingham Trent University, Southwell, United Kingdom

**Keywords:** fats, emulsifiers, growth, footpad lesions

## Abstract

Supplementation of a combination of lysolecithin, a synthetic emulsifier, and monoglycerides (**LEX**) in liquid and dry form to broiler diets with different energy levels was investigated to determine their effect on performance, litter quality and subsequent occurrence of footpad lesions. One thousand two hundred and forty-eight-day-old Ross 308 broilers were assigned to 1 of 6 treatments for a 42-day study: a basal diet with a normal energy content (**NE**); NE + 300 g/t LEX in liquid form (**LEL**); NE + 500 g/t LEX in dry form (**LED**); a basal diet with low energy (LE, −90 kcal/kg starter, −100 kcal/kg grower, finisher), LE + 300 g/t LEL and a LE + 500 g/t LED. Each treatment consisted of 13 pens of 16 birds each. Diets were fed in 3 phases (starter d 0–10, grower d 11–21, finisher d 22–42). Feed intake and weight were measured on d 0, 10, 21, and 42. On d 42 a litter sample was collected from each pen and 2 birds per pen were assessed for footpad lesions and breast scald. Data were analyzed using JMP 16, with means separation achieved using Tukey's HSD; significance was assumed at *P* < 0.05. Results showed a higher (*P* < 0.05) cumulative bodyweight gain with LEX supplementation (NE CON = 2,718 g, NE+LED = 2,829, NE+LEL = 2,895, LE CON = 2,722, LE+LED = 2,787, LE+LEL = 2,893; *P* = 0.0027). An increased feed intake was observed for the LE diets, however cumulative FCR of LE+LED and LE+LEL remained equal to the NE control (1.657 NE CON, 1.657 LE+LED, 1.623 LE+LEL; *P* > 0.05), suggesting LEX enabled the birds to compensate for the energy gap. Litter dry matter was significantly improved with both LED and LEL supplementation compared to the control groups, and resulted in lower (*P* < 0.05) occurrence and severity of footpad lesions and breast scalds. Considering the income over feed cost (**IOFC**) of the NE treatment as the reference point for comparison, all other treatments improved profitability, with NE+LEL and LE+LEL achieving the greatest IOFC with 154.58 and 175.96 €/1,000 birds respectively. In conclusion, feeding broilers a combination of lysophospholipids, a synthetic emulsifier and monoglycerides resulted in improved bird performance. The use of the LEX also improved litter quality and footpad health, therefore improving animal welfare indicators such as breast scald and footpad measurements.

## INTRODUCTION

Excess undigested fats at the end of the intestine not only cannot contribute to broiler growth and performance, but can contribute to increased faecal moisture (Collett, 2012) and the development of wet litter. Wet litter in commercial broilers is linked to increased NH_3_ emissions, disease outbreaks, reduced weight gain and increased welfare issues including the development of lameness, sores, lesions and feather pecking (Zampiga et al., 2016).

Previous studies have shown that nutrient absorption enhancers based on lysolecithin are effective tools to improve dietary energy and protein availability (Boontiam et al., 2019)—especially when supplemented in combination with other potentiating compounds such as synthetic surfactants and monoglycerides (**LEX**, Wealleans et al., 2020a, b). LEX is also able to contribute directly to the emulsion and hydrolysis of dietary fats (Michels et al., 2023), thereby releasing other nutrients from the fat matrix. Furthermore, lysolecithin is known to become incorporated into the phospholipid bi-layers of cells, inducing local curvatures and alterations in protein channel formation, thereby improving the fluidity and permeability of the membrane (Lundbaek and Andersen, 1994) and inducing epigenetic alterations related to collagen deposition (Brautigan et al., 2017). LEX supplementation results in improved villus height (Brautigan et al., 2017; Ghazalah et al., 2021) and increased absorptive capacity, driving improvements in nutrient digestibility beyond lipid absorption (Wealleans et al., 2020a). These nonlipid-related effects may explain recent work by Ghazalah et al. (2021), which showed that the ability of LEX to improve feed efficiency and growth, both in “on top” and reformulated diets, is not only linked to the utilization of added fat in high-density diets, but can also improve the performance and profitability of birds fed low-energy diets containing no added fat.

Previous studies have focused on the use of LEX as a dry powder, due to ease of transport, handling, and application. However, it was hypothesized that liquid delivery might enable a more rapid, stable, and homogenous distribution of the active ingredients throughout the feed, though no published data are currently available to support this hypothesis. The present study aimed to compare LEX supplementation as either a dry or liquid formulation, on the growth performance, nutrient digestibility, litter moisture and selected welfare parameters of broilers fed either normal or low energy diets.

## MATERIALS AND METHODS

The accommodation and care of animals used in this study was in accordance with Directive 2010/63/EC and European Commission Recommendation 2007/526/EC. The protocol and all procedures used in this experiment were approved by the Kemin Industries review process (KAE-21-46).

The trial was conducted at the facilities of Roslin Nutrition, UK. One thousand two hundred and forty-eight-day-old male Ross 308 broilers were procured from P.D Hook Hatchery, Cote, Oxfordshire. All birds were individually weighed on arrival, then randomly assigned to 6 dietary treatments with 13 pen replicates of 16 birds per treatment.

Birds were raised in a thermostatically controlled room, following a commercial temperature program. Lighting provided with 1 h of darkness from d 1 increasing by 1 h per d to d 6, and subsequently 6 h darkness in a 24-h period, with one 4-h block, after the first 7 d. Birds were bedded on wood shavings and mortality was recorded daily throughout the trial.

Two basal control diets were formulated: a normal energy (**NE**) control, formulated to meet Aviagen nutrient requirements, and a low energy (**LE**) control with a 90 to 100 kcal reduction in energy, dependent on phase, compared to the NE. This downspec is based on previous commercial and academic experience (Ghazalah et al., 2021). NE and LE diets were isonitrogenous and provided balanced mineral profiles. A further 4 experimental diets were created by the addition of a combination of lysolecithin, synthetic emulsifier and monoglycerides (Lysoforte EXTEND, Kemin Europa N.V., Herentals, Belgium; LEX) in either dry or liquid form to both the NE and LE controls. The dry formulation (**LED**) contained the active ingredients on a limestone carrier, and was added to the feed at 500 g/t, the standard commercial rate of supplementation. The liquid formulation (**LEL**) contained the active ingredients in a rapeseed oil carrier and was added to the feed at 300 g/t. The experimental treatments were therefore: i) NE, ii) NE+LED, iii) NE+LEL, iv) LE, v) LE+LED, vi) LE+LEL.

The composition of the experimental diets is shown in Table 1. They were fed in 3 phases, with a starter diet from 0 to 10 d, a grower diet from 11 to 21 d, and a finisher diet from d 22 to 42. All diets contained 500 FTU/kg of a commercial phytase. Diets were manufactured by Roslin Nutrition and fed as mash. Feed and water were provided ad libitum throughout the trial.

**Table 1:** Ingredients, calculated nutrient composition (as fed), and costs of the normal (NE) and low energy (LE) diets used in this study.^1^

	Starter (d 0–10)		Grower (d 11–21)		Finisher (d 22–42)	
Feed composition	NE	LE	NE	LE	NE	LE
Ingredients (%)						
Corn	46.882	45.721	48.325	48.909	51.819	52.307
Wheat	10.00	10.00	10.00	10.00	10.00	10.00
Rapeseed meal 35% CP	-	3.50	-	4.23	-	4.33
Soybean meal 47% CP	35.27	35.21	32.49	31.05	30.43	28.63
Full-fat soya 35% CP	1.50	-	2.40	-	2.00	-
Soya oil	2.40	1.80	3.00	2.10	3.14	2.20
MCP	0.92	0.88	0.68	0.65	0.45	0.42
Sodium bicarbonate	0.30	0.28	0.18	0.19	0.10	0.10
Limestone	1.19	1.17	1.18	1.13	0.90	0.85
Sodium chloride	0.27	0.27	0.30	0.29	0.30	0.30
DL-methionine	0.333	0.30	0.290	0.271	0.227	0.207
L-lysine HCl	0.233	0.208	0.144	0.166	0.084	0.106
L-threonine	0.116	0.097	0.082	0.083	0.040	0.040
L-valine	0.076	0.054	0.019	0.021	-	-
Phytase 5000	0.010	0.010	0.010	0.010	0.010	0.010
Vit-min premix^2^	0.500	0.500	0.500	0.500	0.500	0.500
Titanium dioxide	-	-	0.400	0.400	-	-
Calculated nutrients						
AME (kcal/kg)	2885	2795	2960	2860	3010	2910
Crude protein (%)	22.50	22.50	21.00	21.00	20.00	20.00
Fat (%)	5.06	4.25	5.85	4.64	6.00	4.81
Crude fiber (%)	3.20	5.63	3.14	3.45	3.06	3.38
Dig. lysine (%)	1.22	1.22	1.10	1.10	1.00	1.00
Dig. Met (%)	0.63	0.61	0.57	0.56	0.50	0.49
Dig Met + Cys (%)	0.90	0.90	0.84	0.84	0.76	0.76
Dig. Thr (%)	0.79	0.79	0.73	0.73	0.66	0.66
Dig. Ile (%)	0.82	0.83	0.78	0.77	0.75	0.73
Dig. Val (%)	0.96	0.96	0.87	0.87	0.82	0.81
Dig. Arg (%)	1.34	1.36	1.28	1.25	1.21	1.19
Dig. Trp (%)	0.23	0.24	0.22	0.22	0.21	0.21
Calcium (%)	0.95	0.96	0.90	0.90	0.75	0.75
Total P (%)	0.58	0.60	0.52	0.54	0.46	0.48
Dig. P (%)	0.45	0.45	0.40	0.40	0.35	0.35
Sodium (%)	0.19	0.19	0.17	0.17	0.15	0.15
Cost (€/t)	506.13	489.46	505.84	482.09	497.33	474.02
Analyzed nutrients						
Dry matter, %	85.7	87.9	86.9	87.9	87.6	88.1
Oil A, %	5.0	4.4	5.3	4.2	6.0	4.4
Crude protein, %	20.3	20.1	20.4	20.1	19.7	19.9
Ash, %	5.0	5.4	5.4	5.4	4.6	4.6
Calcium, %	0.72	0.76	0.74	0.76	0.66	0.60
Phosphorus, %	0.53	0.53	0.52	0.53	0.46	0.46

1 To create treatment diets LYSOFORTE EXTEND DRY or LYSOFORTE EXTEND LIQUID (a combination of lysolecithin, synthetic emulsifier, and monoglycerides manufactured by Kemin Europa NV, Herentals, Belgium) were added at 500 or 300 g/t at the expense of corn.

2 Provided per kilogram diet: vitamin A: 12,000 IU; vitamin D3: 2,400 IU; vitamin E: 30 mg; vitamin K3: 3 mg; vitamin B1: 2.2 mg; vitamin B2: 8 mg; vitamin B6: 5 mg; vitamin B12: 11 µg; folic acid: 1.5 mg; biotin: 150 mg; calcium pantothenate: 25 mg; nicotinic acid: 65 mg; Mn: 60 mg; Zn: 40 mg; I: 0.33 mg; Fe: 80 mg; Cu: 8 mg; Se: 0.15 mg; ethoxyquin: 150 mg, 12,000 IU.

Feed intake (**FI**) and bird weight (pen basis) were measured on a weekly basis. Pen weight was divided by the number of birds allowing an average body weight gain (**BWG**) to be recorded. Feed conversion ratio was calculated by phase and overall, by dividing the average feed intake by the average total weight gain.

Litter moisture was assessed by collecting litter samples from each pen on d 42 of the trial, with litter from the center and each corner of the pen; samples were not collected from areas immediately below or adjacent to drinkers. Dry matter percentage was assessed by weighing then placing each sample in a forced air oven at 105°C until they achieved a constant weight. Dried samples were reweighed, following cooling to room temperature in a desiccator.

Footpad and breast scald scoring was performed on d 42, when 2 birds per pen were randomly selected and scored. A score of zero was given if no lesion was present, a score of 1 indicated a minor lesion was present and a score of 2 indicated a severe lesion. For breast scald measurements, a score of zero was given if no skin irritation was present. A score 1 indicated feather loss and mild irritation, while a score of 2 indicated substantial feather loss, red skin irritation or broken skin.

On d 21, 4 birds/pen were randomly selected and euthanized by cervical dislocation. The digesta from the ileum (the section from Meckel's diverticulum to the cecal tonsil junction) were immediately collected and pooled per pen, rapidly frozen on dry ice, and stored at −20°C before analysis. All digestibility analyses were conducted by DM Scientific (Dalton, UK). Briefly, grower feed and ileal samples were ground into fine powder and AOAC methods were used to measure the contents of dry matter, TiO_2_, crude protein (N × 6.25), crude fat, and starch, as per Craig et al. (2020). Digestibility coefficients were calculated using the following equation:

$$\text{Digestibility}\;\left(\%\right)=\;\left[1-\;\left(\frac{Ti_{\text{diet}}}{Ti_{\text{il}}}\right)\;\times \;\left(\frac{\text{Nut}r_{\text{il}}}{\text{Nut}r_{\text{diet}}}\right)\;\times \;100\;\right]$$

where Ti_diet_ and Nutr_diet_ represent TiO_2_ and nutrient contents in feed samples, respectively, while Ti_il_ and Nutr_il_ reflect the contents of TiO_2_ and nutrients in collected ileal samples.

To compare the economic impact of the dry and liquid applications of the combination of lysolecithin, synthetic emulsifier and monoglycerides to normal or low energy diets, a cost-benefit calculation was made based on real performance data, the weighted average of feed cost based on real feed intake. The model assumed no difference in body weight gain (**BWG**) between treatment groups. Economic parameters assumed representative market prices at time of trial, based on actual costs of the raw ingredients. Net benefit at the farmgate was calculated using a conventional broiler live weight price of 1.3 €/kg, and presented as income over feed cost (**IOFC**, €/1,000 birds).

For statistical analysis, the pen/replicate was considered the experimental unit. No outlier data were identified or excluded from the dataset. Data were analyzed using the Fit Model capability of JMP 16 (SAS Institute, Cary, NC), as a 2 × 3 factorial with dietary energy level (NE, LE) and lysolecithin supplementation (none, LED, LEL) as the main factors. Furthermore, Tukey's HSD was used to assess means separation across all individual treatments (NE, NE+LED, NE+LEL, LE, LE+LED, LE+LEL). Differences were considered significant at *P* < 0.05; *P* < 0.1 was taken to indicate a near-significant trend.

## RESULTS AND DISCUSSION

The ability of combination nutrient absorption enhancers based on lysolecithin to improve nutrient digestibility and absorption, thereby driving improved broiler growth performance and profitability is well documented (Wealleans et al., 2020a, b; Ghazalah et al., 2021). However, to the authors’ knowledge, no previous studies have directly assessed the impact of formulation on the outcomes of supplementing broiler diets with nutrient absorption enhancers based on lysolecithin. The results of the present study are summarized in Table 2.

**Table 2:** Effect of the addition of a combination of lysolecithin, synthetic emulsifier and monoglycerides in dry (LED) or liquid (LEL) formulations to normal or low energy diets on broiler performance.

	Treatment						*P* value		
Parameter	NE	LE	CON	LED	LEL	SEM	Energy	LEX	Energy*LEX
Performance									
BW d 0 (g/bird)	43.65	43.55	43.47	43.90	43.45	0.406	0.7759	0.4970	0.2730
BW d 10 (g/bird)	267.52	270.34	262.58b	269.76ab	274.70a	3.255	0.2842	0.0034	0.9941
BWG 0–10 (g/bird)	223.87	226.80	219.11b	225.87ab	231.26a	3.167	0.2559	0.0026	0.9877
FI d 0–10 (g/bird)	277.52	275.22	271.69	274.22	283.99	5.407	0.6302	0.0863	0.2257
FCR d 0–10	1.241	1.214	1.241	1.213	1.230	0.020	0.1062	0.4147	0.0851
BW d 21 (g/bird)	921.46	910.14	909.28	913.52	924.89	7.913	0.0960	0.1671	0.0934
BWG d 10–21 (g/bird)	653.94	639.80	646.71	643.76	650.19	7.841	0.0352	0.7417	0.0977
FI d 10–21 (g/bird)	905.96b	925.69a	904.92	916.95	926.75	9.890	0.0217	0.1220	0.3790
FCR d 10–21	1.387b	1.449a	1.400	1.426	1.429	0.017	<0.0001	0.2256	0.0287
BW d 42 (g/bird)	2858.01	2841.88	2763.41b	2852.06ab	2937.09a	61.028	0.8007	0.0332	0.9269
BWG d 21–42 (g/bird)	1936.56	1931.75	1854.13b	1938.54ab	2012.21a	58.075	0.9714	0.0435	0.8559
FI d 21–42 (g/bird)	3361.55	3464.19	3354.61	3399.51	3486.24	61.568	0.0529	0.1319	0.7230
FCR d 21–42	1.740b	1.811a	1.829a	1.756ab	1.738b	0.034	0.0237	0.0356	0.8719
BWG d 0–42 (g/bird)	2814.37	2798.34	2719.95b	2808.17ab	2893.65a	60.994	0.8021	0.0331	0.9242
FI d 0–42 (g/bird)	4545.02b	4665.09a	4531.21	4590.68	4696.97	67.771	0.0400	0.0737	0.8376
FCR d 0–42	1.617b	1.674a	1.674x	1.636xy	1.626y	0.021	0.0027	0.0747	0.9560
Digestibility									
Dry matter, %	60.98b	68.73a	63.75	66.47	64.35	2.537	0.0222	0.1220	0.8893
Crude Protein, %	74.02a	64.24b	68.54	70.60	68.26	2.513	0.0092	0.0451	0.8356
Gross energy, %	84.57	86.00	83.43x	85.83xy	86.61y	1.416	0.3188	0.0899	0.1394
Litter moisture and welfare indicators									
Litter DM, %	59.95	61.7	50.03^b^	66.21^a^	66.22^a^	1.4280	0.2927	<0.0001	0.6574
Footpad lesions	1.00	1.13	1.69^a^	0.77^b^	0.73^b^	0.0098	0.1123	<0.0001	0.6656
Breast scald	1.23	1.22	1.71^a^	0.96^b^	1.00^b^	0.1173	0.8937	<0.0001	0.2734

Across the whole study, application of both LED and LEL resulted in significantly increased body weight gain (NE CON = 2,718, NE+LED = 2,829, NE+LEL = 2,895, LE CON = 2,722, LE+LED = 2,787, LE+LEL = 2,893; *P* = 0.0331). There was no significant effect of energy level (*P* = 0.8021) or interaction between energy level and LEX supplementation (*P* = 0.9242). The cumulative feed intake results showed a significant difference in feed intake (*P* = 0.04) between treatments for the associated energy level, with the low energy treatments consuming significantly more feed. FCR results revealed a significant effect between treatments for the energy level of the diets (NE CON = 2,718, NE+LED = 2,829, NE+LEL = 2,895, LE CON = 2,722, LE+LED = 2,787, LE+LEL = 2,893; *P* = 0.0027), with the LE treatments having significantly increased feed conversion ratios. Likewise, there was a statistical trend toward the LEX treatments having improved FCRs compared to the untreated controls (*P* = 0.0747).

The addition of the lysolecithin combination in any formulation significantly reduced litter moisture compared to the control diets (*P* < 0.05), as shown in Table 2. There was no effect of energy density on litter moisture, and no interaction between energy and LEX supplementation. Litter dry matter was numerically increased in pens fed diets containing LEL compared to LED, with increases of 2.64 and 2.77% in normal and low energy diets, respectively.

Performance improvements following LEX supplementation are often driven by improvements in nutrient digestibility and retention (Wealleans et al., 2020a; Ghazalah et al., 2021). In the present study, there were significant effects of energy level on dry matter and crude protein digestibility, with low energy diets reporting lower digestibility coefficients. When analyzed factorially, there was also a significant effect of LEX addition (74.06% LEL, 73.79% LED, 68.25% CON, *P* = 0.0451). A similar pattern was seen for dry matter digestibility, where there was a near-significant trend in favor of the LED and LEL treatments. ME digestibility was not affected by dietary energy density, though again there was a near-significant trend in favor of the LED and LEL treatments overall (83.42% CON, 85.83% LED, 86.61% LEL, *P* = 0.0899).

Litter moisture levels are linked to broiler gut health status and are known to be driven in part by the extent of fat digestion: Collett (2012) postulated that high excreted fat levels had both direct (increasing fecal moisture) and indirect (reducing litter water holding capacity) effects on litter moisture. In the present study, addition of LEX in both formulations significantly reduced the severity of footpad lesions and breast burns: LED and LEL in NE diets resulted in an 86 and 82% reduction in footpad score, and a 57 and 38% reduction in severity of breast burns. In LE diets, LED and LEL lead to reductions of 65 and 68% in footpad lesions and 56 and 67% in breast burns, respectively. The difference between supplemented and unsupplemented diets was significant (*P* < 0.0001 footpad lesions, *P* < 0.0001 breast burns) in all cases, but there was no significant effect of energy level or difference between LEX formulations. This is perhaps contrary to initial indications, as the LE diets contained lower levels of dietary fats, which should naturally reduce lipid excretion into the litter (Collett, 2012).

Taken together, improved nutrient digestibility is the likely driver of improved performance following lysolecithin supplementation. Though the difference between LEX formulations on performance was not statistically significant, a number of numerical trends could be clearly observed: supplementation of LED increased BW by 3.87 and 2.53% in NE and LE diets, while LEL increased by 6.22 and 6.34% in NE and LE diets. Similarly, LED reduced FCR by 3.98 and 2.93% in NE and LE diets, while the reduction with LEL was 5.61 and 4.92%.

Improved FCR, often linked to improved health and welfare, is a major driver of increased profitability in broiler production, and in the present study, considering the IOFC of the normal energy control treatment as the reference point for comparison, all other treatments provided better financial results. Profitability as measured by IOFC (€/1,000 birds) was clearly different by treatment (NE CON = 1,296.23, NE+LED = 1,397.07, NE+LEL = 1,450.81, LE CON = 1,353.78, LE+LED = 1,403.28, LE+LEL = 1,472.19). The best profitability was seen for LEL supplementation in an LE diet, which earned 175.96 and 118.41 €/1,000 birds more than NE and LE controls, respectively. LEL supplementation in an NE diet was the next most profitable application, earning 154.58 and 97.03 €/1,000 birds more than NE and LE controls. Application of LED earned between 43 and 107 €/1,000 birds more than control diets, depending on control and comparison.

In conclusion, the present study investigated the impact of lysolecithin-based nutrient absorption enhancers, assessing both dry and liquid formulations, on broiler performance, limited welfare parameters, and profitability. The research confirmed the efficacy of lysolecithin in enhancing nutrient digestibility, resulting in improved body weight gain and feed efficiency, especially notable in the liquid formulation. Both formulations significantly reduced litter moisture, footpad lesions, and breast burns. The study highlighted that regardless of the delivery mechanism, LEX substantially enhanced nutrient absorption and retention, boosting broiler performance, and also contributed to reduce litter moisture. Economic analysis revealed that both dry and liquid formulations led to increased profitability compared to nonsupplemented diets, with the liquid formulation showcasing slightly enhanced financial benefits. While this study showed numerical advantages for the liquid formulation, it established that supplementing broiler diets with LEX, regardless of formulation, significantly improves profitability and bird welfare. Further investigations could explore optimization of the liquid formulation for maximal benefits in broiler production.

## DISCLOSURES

The authors declare the following financial interests/personal relationships which may be considered as potential competing interests: Alexandra Wealleans reports article publishing charges was provided by Kemin Europa NV. Kemin Europa NV funded the study, and provided support in the form of salary for A. W., A. D., D. G. S., and R. S. No other conflicts of interest are declared.
